# Intersecting substance use treatment and harm reduction services: exploring the characteristics and service needs of a community-based sample of people who use drugs

**DOI:** 10.1186/s12954-022-00676-8

**Published:** 2022-08-24

**Authors:** Noa Krawczyk, Sean T. Allen, Kristin E. Schneider, Keisha Solomon, Hridika Shah, Miles Morris, Samantha J. Harris, Susan G. Sherman, Brendan Saloner

**Affiliations:** 1 Center for Opioid Epidemiology and Policy, Department of Population Health, NYU Grossman School of Medicine, New York, USA; 2 Department of Health, Behavior, and Society, Johns Hopkins Bloomberg School of Public Health, Baltimore, USA; 3 Department of Health Policy and Management, Johns Hopkins Bloomberg School of Public Health, Baltimore, USA

**Keywords:** Harm reduction, Treatment, Opioid use disorder, Health services research, Syringe service programs, Methadone, Buprenorphine, Low threshold, Service integration

## Abstract

**Background:**

Substance use treatment and harm reduction services are essential components of comprehensive strategies for reducing the harms of drug use and overdose. However, these services have been historically siloed, and there is a need to better understand how programs that serve people who use drugs (PWUD) are integrating these services. In this study, we compared treatment and harm reduction services offered by a multistate sample of substance use service providers and assessed how well they align with characteristics and needs of clients they serve early in the COVID-19 pandemic.

**Methods:**

We recruited a convenience sample of programs that deliver harm reduction and/or treatment services in ten US states. Program directors participated in a survey assessing the services offered at their program. We also recruited clients of these programs to participate in a survey assessing a range of sociodemographic and health characteristics, substance use behaviors, and health service utilization. We then cross-compared client characteristics and behaviors relative to services being offered through these programs.

**Results:**

We collected and analyzed data from 511 clients attending 18 programs that we classified as either offering treatment with medications for opioid use disorder (MOUD) (*N* = 6), syringe service programs (SSP) (*N* = 8), or offering both MOUD and SSP (*N* = 4). All programs delivered a range of treatment and harm reduction services, with MOUD & SSP programs delivering the greatest breadth of services. There were discrepancies between services provided and characteristics and behaviors reported by clients: 80% of clients of programs that offered MOUD without SSP actively used drugs and 50% injected drugs; 40% of clients of programs that offered SSP without MOUD sought drug treatment services. Approximately half of clients were unemployed and unstably housed, but few programs offered direct social services.

**Conclusions:**

In many ways, existing programs are not meeting the service needs of PWUD. Investing in innovative models that empower clients and integrate a range of accessible and flexible treatment, harm reduction and social services can pave the way for a more effective and equitable service system that considers the long-term health of PWUD.

## Background

Substance use treatment and harm reduction services are essential components of comprehensive strategies for mitigating harms associated with drug use and overdose. Traditionally, these services have been perceived to have distinct goals, with treatment aiming to reduce or eliminate drug use, and harm reduction aiming to minimize health risks for people who use drugs (PWUD), including those driven by the criminalization of drug use. These goals are not incompatible and represent a spectrum of options that may benefit and meet the needs of PWUD at different moments; however, these services have been historically siloed due to cultural and structural factors. In the USA and many other parts of the world, the punitive “War on Drugs” created an addiction treatment system that heavily valued abstinence and criminalization of drug use and drug users, especially from minoritized groups, and left little room to accept or even acknowledge ongoing drug use [1]. In response to the Drug War and the rise of HIV/AIDS, the Harm Reduction Movement, led mainly by PWUD aiming to empower and protect their health, rights and realities, made a concerted effort to dissociate its philosophy and programs from the narrative of abstinence as a necessary goal. Instead, the Harm Reduction Movement focused on offering services such as syringe services programs (SSPs) that can reduce the risk of infectious disease and other drug-related harms to people in active use [2].

In the USA, in particular, treatment and harm reduction services have been further divided by distinct regulatory and financing systems. Historically excluded from mainstream healthcare systems and insurance parity, substance use treatment programs became operated by a mix of for-profit and non-profit organizations funded and regulated by government entities [3]. Harm reduction programs, on the other hand, were excluded from both the healthcare and substance use treatment systems; as a result, they have been largely operated and financed by grassroots advocacy and non-governmental organizations [4].

While cultural and structural differences continue to divide many substance use treatment and harm reduction services, the needs and goals of people who seek these two services may have always been much less distinctive. For example, many who attend substance use treatment continue to use drugs [5]. Similarly, many who attend harm reduction programs seek to engage in treatment at some points [6]. Indeed, clients of SSPs are approximately five times more likely to engage in treatment and three times more likely to stop using drugs than persons who do not access SSPs [7]. In recent decades, harm reduction and treatment goals have become increasingly blurred with the growing uptake of medications for opioid use disorder (MOUD). In particular, methadone and buprenorphine are used by some with a goal of abstaining from opioid use; for others, MOUD are used to help mitigate withdrawal and overdose risk without abstaining from drug use [8, 9].

Despite this reality, programs that successfully combine treatment and harm reduction services and principles are often the exception rather than the rule [8, 10, 11]. Yet, the increasing severity of the opioid overdose crisis in North America and the rise in viral and bacterial infections among PWUD [12–14] have led to a recognition of the urgent need to utilize multiple approaches toward the joint goal of reducing drug-related harms [15]. In particular, concerns about the increasingly lethal opioid supply [16] have emphasized the need to use any available evidence-based strategies known to reduce opioid-related overdose mortality. These concerns have encouraged more treatment providers to incorporate harm reduction approaches (e.g., naloxone distribution and overdose education) [17], and harm reduction providers to integrate MOUD as a direct service [18].

Thus, there is a need to better understand how existing drug treatment and harm reduction programs integrate services and how well they align with client characteristics, needs and behaviors. Most current health services research has focused separately on questions of delivery and access to treatment or harm reduction services, rather than their integration. In this paper, we aim to contribute to the paucity of literature examining the integration of these services by analyzing data from a sample of clients recruited from diverse programs serving PWUD in ten US states. While our sample includes individuals who seek treatment or harm reduction services regardless of drugs used, given the high prevalence of opioid use in the USA we specifically compared characteristics across programs that provide more traditional treatment for opioid use (i.e., with MOUD), harm reduction services (i.e., SSPs), and those that combine both strategies (e.g., MOUD & SSPs). By assessing client characteristics and behaviors relative to the features and services provided by the programs with which they engage, we aim to identify service gaps and discuss implications for a comprehensive substance use service system that more effectively meets the needs and promotes the health of PWUD.

## Methods

### Participant recruitment

This analysis is part of a larger study that aimed to collect data on drug use behaviors and health service utilization during the COVID-19 pandemic in US states that participate in the Bloomberg Overdose Initiative to reduce overdose across the country. With the help of local partners of the initiative, the study team recruited a convenience sample of 22 programs that deliver treatment and/or harm reduction services to PWUD in ten states, including Washington, D.C., Maine, Maryland, Michigan, New Jersey, New Mexico, New York, Pennsylvania, Tennessee, and West Virginia. One person from each participating program (usually a member of the program’s leadership, e.g., Program Directors) was recruited to participate in a “provider survey” that included questions about the range of services offered at their program, general characteristics of clients, and responses to the changing circumstances caused by the COVID-19 pandemic. Providers were then asked to assist in recruitment of their clients for a “client survey,” which inquired about substance use behaviors and service utilization prior to and during the COVID-19 pandemic, by distributing recruitment cards to clients. Data collection for both provider and client surveys occurred between August 2020 and January 2021. Study protocols and survey instruments were reviewed by an external advisory board comprised of service providers, organizations representing PWUD, and national substance use experts. The Johns Hopkins Institutional Review Board approved this study.

### Provider survey

Provider surveys were conducted with trained interviewers over the phone or self-conducted online via Qualtrics software (accessed through a link that was sent to program contacts during recruitment). Only one survey was completed for each participating program, and the completing participant provided informed consent. Providers took approximately 20 min to complete the survey and were compensated $40 upon survey completion. Survey items included questions about the organization at which they worked, including size of staff and clients, general sociodemographic characteristics of clients served, and which services were and were not offered at their program (including a range of services labeled as “harm reduction,” “substance use treatment,” “social and wraparound services,” and “medical care”). Survey questions also asked about the COVID-19 pandemic; specifically, we wanted to know about any challenges with service provision and the extent to which service availability changed prior to and after the pandemic. Although these findings are not the focus of the current paper, they are reported elsewhere along with more details about the study procedures [19].

### Client survey

Clients were recruited for the client survey using 100–150 recruitment cards mailed to each participating program. Each recruitment card included the study logo with the study phone number, and business hours during which clients could call to participate in the survey. Each card displayed a unique identifying code to reduce repeat interviews and to ensure recruitment of individuals directly engaged in services. Clients who called the phone line spoke with trained interviewers who screened them for study eligibility (at least 18 years old; currently a client of a referring harm reduction or treatment organization; able to provide informed consent and a unique study identifier). The survey was then conducted via phone and lasted approximately one hour. Clients who completed the interview received $40 dollar compensation. Survey items asked participants about a range of sociodemographic and health characteristics, substance use behaviors (including drug types, frequency, and modes of use), drug use safety practices, overdose experiences, and interaction with a range of health services (including treatment and harm reduction services). Items which asked about changes in behaviors and service utilization prior to and during COVID-19 are explored in other analyses [19].

### Data analysis

For the purposes of this analysis, we classified participating programs into three mutually exclusive categories based on the following services they offered (as indicated in the provider survey): (1) Programs offering treatment with medications for opioid use disorder (MOUD) but no syringe services program (SSP) “MOUD”; (2) programs offering treatment with MOUD and SSP services “MOUD & SSP”; and (3) programs offering SSP services but no treatment with MOUD “SSP.” We selected this categorization in order to enable comparison between more traditional harm reduction and treatment services. While not all clients in our sample used opioids, the majority did and we therefore excluded one treatment program that did not offer evidence-based medications for OUD (a recovery support center). We also excluded two-community-based programs and one health department program that offered some overdose prevention services and resources but no SSP or MOUD. Our final sample included 18 programs from which 511 clients participated in surveys. We compared differences in select characteristics and services provided across program types using Fisher’s exact test for categorical variables to account for small cell sizes, and ANOVA tests for continuous variables. To compare client characteristics among program types (while accounting for client clustering within programs), we used linear, logit, and multinomial regression models with a random effect for the program from which clients were recruited. Statistically significant differences were identified if the p-value was less than 0.05. All analyses were conducted in Stata version 15 [20].

## Results

### Program characteristics

We analyzed data from 18 programs including six that offered only MOUD, four that offered both MOUD & SSP, and eight that offered only SSP. Table 1 compares select characteristics across the three program types, noting differences in attributes that were statistically significant among them. Most programs (89%) identified as non-profit organizations. The mean size of the program staff was greatest in MOUD (58.3), followed by MOUD & SSP (36.8) and SSP programs (18.8).

**Table 1:** Characteristics of Providers (*N* = 18)

	Total	MOUD only (*N* = 6)	MOUD & SSP (*N* = 4)	SSP only (*N* = 8)	*P* value
Organization type					0.50
Non-profit organization	16 (88.9%)	6 (100%)	3 (75%)	7 (87.5%)	
City or state-run program	1 (5.6%)	0 (0%)	0 (0%)	1 (12.5%)	
Univ/medical organization	1 (5.6%)	0 (0%)	1 (25%)	0 (0%)	
Workforce size (Mean [Std Deviation])	35.9 [37.9]	58.3 [48.3]	36.8 [43.8]	18.8 [15.6]	0.16
Harm reduction services					
*Syringe service program*	12 (66.7%)	–	4 (100%)	8 (100%)	–
Overdose prevention/education	16 (88.9%)	4 (66.67%)	4 (100%)	8 (100%)	0.14
Naloxone distribution	16 (88.9%)	4 (66.67%)	4 (100%)	8 (100%)	0.14
Fentanyl testing/test-strip distribution	12 (66.7%)	2 (33.3%)	3 (75%)	7 (87.5%)	0.16
Drug checking machine	1 (5.6%)	0 (0%)	0 (0%)	1 (12.5%)	1.00
Peers/street outreach for harm reduction	15 (83.3%)	3 (50%)	4 (100%)	8 (100%)	**0.03**
Treatment services					
*Medications for opioid use disorder (MOUD)*	10 (45.5%)	6 (100%)	4 (100%)	–	**–**
Methadone maintenance	5 (27.8%)	5 (83.33%)	0 (0%)	–	0.05
Methadone as detox/taper	3 (16.7%)	3 (50%)	0 (0%)	–	0.20
Buprenorphine maintenance	8 (44.4%)	5(83.33%)	3 (75%)	–	1.00
Buprenorphine as detox/ taper*	4 (22.2%)	1 (16.67%)	3 (75%)	–	0.19
Extended-release naltrexone maintenance	3 (16.7%)	2 (33.3%)	1 (25%)	–	1.00
Individual counseling/behavioral therapy	12 (66.7%)	5 (83.33%)	4 (100%)	3 (37.5)	0.06
Group counseling/behavioral therapy	11 (61.1%)	5 (83.33%)	3 (75%)	3 (37.5)	0.24
Peer recovery coaches	10 (55.6%)	3 (50%)	2 (50%)	5 (62.5%)	1.00
Self-help/12-step programs	3 (16.7%)	1 (16.67%)	1 (25%)	1 (12.5%)	1.00
Urine/saliva drug screening	7 (38.9%)	6 (100%)	1 (25%)	0 (0%)	**< 0.001**
Walk in/same day treatment initiation	9 (50.0%)	3 (50%)	4 (100%)	2 (25%)	0.06
Social and wraparound services					
On-site case management/social services	13 (72.2%)	3 (50%)	3 (75%)	7 (87.5%)	0.35
Referral to case management/social services	14 (77.8%)	6 (100%)	3 (75%)	5 (62.5%)	0.28
Housing units provided by program	4 (22.2%)	1 (16.67%)	1 (25%)	2 (25%)	1.00
Educational services/job training/GED	2 (11.11%)	0 (0%)	0 (0%)	2 (25%)	0.48
Parenting/childcare/pregnancy services	6 (33.3%)	4 (66.67%)	2 (50%)	0 (0%)	**0.02**
Domestic violence services	3 (16.7%)	1 (16.67%)	2 (50%)	0 (0%)	0.07
Drop in area/safe space	8 (44.4%)	0 (0%)	4 (100%)	4 (50%)	**0.01**
Medical services					
Wound care	9 (50.0%)	0 (0%)	4 (100%)	5 (62.5%)	**0.01**
Infectious disease testing and/or treatment	13 (72.2%)	3 (50%)	4 (100%)	6 (75%)	0.27

*P* values bolded for significant comparisons across groups using Fisher’s exact test and ANOVA < 0.05 or *P* < 0.001

Services designated as “harm reduction” in the provider survey were offered more frequently by SSP or MOUD & SSP programs. All SSP and MOUD & SSP programs offered overdose prevention and naloxone distribution with harm reduction peer/street outreach; most offered fentanyl test strips (88% and 75%, respectively, in SSP and MOUD & SSP programs). In contrast, only 67% of MOUD programs offered overdose prevention or naloxone distribution, 50% offered some kind of harm reduction street outreach, and 33% offered fentanyl test strips.

Services designated as “substance use treatment” in the survey were offered more frequently by MOUD and MOUD & SSP programs. Methadone maintenance and taper were only offered in MOUD programs (respectively, 83% and 50%). Buprenorphine maintenance was offered by most MOUD (83%) and MOUD & SSP (75%) programs. Buprenorphine taper was offered more often in MOUD & SSP (75%) relative to MOUD programs (17%), potentially indicating greater use of buprenorphine for withdrawal management in addition to maintenance treatment in these settings. Individual and group counseling was available at most MOUD (both 83%) and MOUD & SSP programs(respectively, 100% and 75%), but less often in SSP programs (both 38%). Programs in each category offered varying peer recovery services (50% of MOUD and MOUD & SSP; 63% of SSP programs). All MOUD programs conducted urine/saliva drug screening (compared to 25% MOUD & SSP and none of SSP programs). All MOUD & SSP programs offered same-day treatment initiation (compared to 50% of MOUD and 25% of SSP programs).

Of services designated in the survey as “wraparound services” and “medical care,” the most common services offered were on-site case management (50% of MOUD, 75% of MOUD & SSP, and 87.5% of SSPs programs) or referral to case management services (100% of MOUD, 75% of MOUD & SSP, and 63% of SSP programs). Only 17% (*n* = 1) of MOUD and 25% of MOUD & SSP and SSP programs offered direct housing units. No MOUD or MOUD & SSP programs offered any education or job training programs. 67% of MOUD programs offered parenting, childcare/pregnancy services, but only 17% (*n *= 1) offered domestic/intimate partner violence programming; half of MOUD & SSP and SSP programs offered both of these types of services. While all MOUD & SSP programs offered a drop-in area, only half of the SSP and none of the MOUD programs offered this service. Wound care and infectious disease testing/treatment services were available in most SSP programs (63% and 75%, respectively), all MOUD & SSP programs, and 50% of MOUD programs.

### Client characteristics

Of the 511 clients surveyed, 245 were from MOUD programs, 94 from MOUD & SSP programs, and 172 from SSP programs. Table 2 displays client characteristics in the three program types, noting characteristics that were statistically significantly different across groups. Clients throughout all programs were split almost evenly between men and women, with the exception of one SSP client and four MOUD & SSP clients identifying as transgender. MOUD clients were somewhat older on average (48 vs. 42 at MOUD & SSP and 44 at SSP programs). While clients of MOUD and MOUD & SSP programs were mostly Black (respectively, 36% and 30%) or Latinx (respectively, 21% and 26%), SSP clients were mostly white (64%). Educational attainment was similar throughout the sample and most clients completed at least high school (or GED). Roughly 17–21% of clients in all three groups were full-time, part-time or seasonally employed. Across all groups, most non-working clients indicated being unemployed, but many (38% of MOUD, 27% of MOUD & SSP and 30% of SSP program clients) were unable to work due to health reasons. Roughly 50% of clients did not report owning or renting their own home, with nearly a third indicated living with family or friends.

**Table 2:** Characteristics of clients (*N* = 511)

	Total	MOUD only (*N* = 245)	MOUD & SSP (*N* = 94)	SSP only (*N* = 172)	*P* value
Gender (*N* = 510)					**< 0.001**
Female	247 (48.43)	115 (46.13%)	44 (46.81%)	88 (51.16%)	
Male	258 (50.59%)	129 (52.87%)	46 (48.94%)	83 (48.26%)	
Transgender	5 (0.98)	0 (0%)	4 (4.26%)	1 (0.58%)	
Mean age (SD) (*N* = 511)	45.59 (11.93)	48.14 (10.79)	42.13 (13.06)	43.85 (12.1)	0.11
Age group (*N* = 511)					**0.04**
18–40	202 (39.53%)	70 (28.57%)	50 (53.19%)	82 (47.67%)	
41–60	245 (47.95%)	143 (58.37%)	31 (32.98%)	71 (41.28%)	
61+	64 (12.52%)	32 (13.06%)	13 (13.83%)	19 (11.05%)	
Race/Ethnicity (*N* = 508)					**< 0.001**
White non-Latinx	213 (41.93%)	102 (41.98%)	2 (41.93%)	109 (63.74%)	
Black non-Latinx	151 (29.72%)	88 (36.21%)	151 (29.72%)	35 (20.47%)	
Latinx, any race	133 (26.18%)	52 (21.40%)	133 (26.18%)	20 (11.70%)	
Other non-Latinx	11 (2.17%)	1 (0.41%)	11 (2.17%)	7 (4.09%)	
Education (*N* = 510)					0.35
Less than high school	149 (29.22%)	85 (34.84%)	31 (32.98%)	33 (19.19%)	
HS degree or GED	216 (42.35%)	96 (39.34%)	38 (40.43%)	82 (47.67%)	
Some college or more	145 (28.43%)	63 (25.82%)	25 (26.50%)	57 (33.14%)	
Employment status (*N* = 511)					**< 0.001**
Employed full, part time, or seasonally	94 (18.40%)	44 (17.96%)	20 (21.28%)	30 (17.44%)	
Unemployed	224 (43.84%)	97 (39.59%)	45 (47.87%)	82 (47.67%)	
Unable to work due to health	168 (32.88%)	92 (37.55%)	25 (26.60%)	51 (29.65%)	
Retired, homemaker, or student	25 (4.89%)	12 (4.90%)	4 (4.26%)	9 (5.23%)	
Housing status (*N* = 510)					0.79
Own or rent home	281 (55.10%)	137 (55.92%)	46 (49.46%)	98 (56.98%)	
Stay w/family or friends	166 (32.55%)	78 (31.84%)	32 (34.41%)	56 (32.56%)	
Shelter/temp housing/motel/street/car/bus/park	49 (9.61%)	23 (9.39%)	12 (12.09%)	14 (8.14%)	
Drug treatment program/jail/prison/other	14 (2.75%)	7 (2.86%)	3 (3.23%)	4 (2.33%)	
Drug use (*N* = 511)					
Used any drugs past month^1^	447 (87.48%)	*204 (83.27%)*	*80 (85.11%)*	*163 (94.77%)*	**0.02**
Used opioids past month^2^	273 (53.42%)	*92 (37.55%)*	*62 (65.96%)*	*119 (69.19%)*	**0.02**
Injected any drugs past month	326 (63.80%)	*139 (56.73%)*	*58 (61.7%)*	*129 (75%)*	0.45
Used any drugs past week^1^	413 (80.82%)	179 (73.06%)	76 (80.85%)	158 (91.86%)	**< 0.001**
Used opioids past week^2^	228 (44.62%)	67 (27.35%)	55 (58.51%)	106 (61.63%)	**0.003**
Injected any drugs past week	296 (57.93%)	127 (51.84%)	*53 (56.38%)*	116 (67.44%)	**< 0.001**
Had OD in past month	49 (9.59%)	11 (4.49%)	10 (10.64%)	28 (16.28%)	**0.003**
Past-month syringe experiences (*N* = 511)					
Went to a syringe exchange	177 (34.64%)	20 (8.16%)	48 (51.06%)	109 (63.37%)	**< 0.001**
Gave someone a needle after using it	24 (4.70%)	5 (2.04%)	0 (0%)	19 (11.05%)	**< 0.001**
Used a syringe after someone	23 (4.50%)	3(1.22%)	0 (0%)	20 (11.63%)	**0.002**
Used cookers/rinse water after someone	48 (9.39%)	5 (2.04%)	6 (6.38%)	37 (21.51%)	**< 0.001**
Gave someone cookers/rinse water after using them?	51 (9.98%)	9 (3.67%)	4 (4.26%)	38 (22.09%)	**< 0.001**
Have syringes confiscated by police?	26 (5.09%)	4 (1.63%)	5 (5.32%)	17 (9.88%)	**< 0.001**
Past-month substance use treatment (*N* = 511)					
Received any substance use treatment	344 (67.32%)	241 (98.37%)	32 (34.04%)	71 (41.28%)	**< 0.001**
Received individual counseling	304 (59.49%)	227 (92.65%)	25 (26.60%)	52 (30.23%)	**< 0.001**
Received group counseling	151 (29.55%)	126 (51.43%)	8 (8.51%)	17 (9.88%)	**0.0002**
Attended self-help groups	97 (18.98%)	70 (28.57%)	12 (12.77%)	15 (8.72%)	**< 0.001**
Received methadone for opioid addiction	277 (54.21%)	225 (91.84%)	15 (15.96%)	37 (21.51%)	**< 0.001**
Received buprenorphine for opioid addiction	38 (5.87%)	8 (3.27%)	8 (8.51%)	14 (8.14%)	0.3
Received vivitrol for opioid addiction	0 (0%)	0 (0%)	0 (0%)	0 (0%)	–
Endorsed perceptions on treatment providers (*N* = 308)					
I am able to get substance use treatment I need	248 (80.52%)	183 (80.26%)	19 (76.0%)	46 (83.64%)	0.41
My provider understands the challenges I am facing	269 (87.34%)	201 (88.16%)	22 (88.0%)	46 (83.64%)	0.65
I have someone to talk to if I have cravings to use	270 (87.66%)	202 (88.60%)	21 (84%)	47 (84.45%)	0.44

*P* values bolded for significant associations between program types and select characteristics < 0.05 or *P* < 0.001

^1^Derived from questions about most recent use of any non-prescribed drugs via swallowing, snorting/sniffing, injection, or smoking

^2^Includes heroin, prescription opioids or fentanyl used alone or in combination with other drugs

Patterns of current drug use were statistically significantly different among the three groups, but past-week drug use (derived from a question about most recent use of any non-prescribed drugs via swallowing, snorting/sniffing, injection, or smoking) was highly prevalent among all groups (73%, 81% and 92%, respectively, across MOUD, MOUD & SSP and SSP groups). Any injection drug use was also common, with 52% of MOUD, 56% of MOUD & SSP, and 67% of SSP clients indicating past-week injection. Past-week opioid use (including heroin, prescription opioids or fentanyl, alone or in combination with other drugs) was lower for MOUD clients (27%) than for MOUD & SSP (59%) and SSP clients (62%).

When asked about receiving substance use treatment in the past month, MOUD program clients were most likely to indicate having received any (98%). However, SSP clients were more likely to indicate receiving any substance use treatment (41%) than those attending MOUD & SSP programs (34%). While nearly all clients of MOUD programs received individual counseling (93%), 27% of MOUD & SSP clients and 30% of SSP clients also indicated participating in this type of service. Group counseling and self-help groups were less common overall and mostly used by clients in MOUD programs. While methadone was primarily used by MOUD clients (92%), MOUD & SSP and SSP clients also indicated some amount of methadone use (16% and 22%, respectively). Conversely, buprenorphine use did not differ statistically among MOUD (3%), MOUD & SSP (9%) and SSP clients (8%). Finally, those who attended treatment were asked whether their needs were met by their providers. Clients in all three program types frequently (> 80%) agreed that they felt that they received the treatment they needed, that providers understood challenges they endure, and that they have someone to talk to in case of drug cravings.

## Discussion

Findings from our study illustrate that many substance use programs do not fit directly into a binary of “harm reduction” or “treatment.” Most of the participating programs in this study reported offering a spectrum of harm reduction and treatment services. Still, SSPs were most likely to offer harm reduction services, MOUD programs were most likely to offer treatment services, and those characterized as offering both MOUD & SSPs were most likely to offer the broadest services. Program clients also did not fit into the supposed binary of “active drug use” vs. “abstinence.” In fact, of the clients who attended MOUD only programs, nearly three quarters reported using non-prescribed drugs in the past week, and more than half reported injecting drugs in the past week; these rates were similar to those reported by clients who attended combined MOUD & SSP programs. Meanwhile, more than 40% of those who attended SSP only programs reported attending some type of drug treatment service in the past month.

Our results reveal some important incongruencies between services being offered by substance use programs and characteristics and behaviors reported by clients who attend such programs*.* For example, while three-quarters of MOUD program clients reported using non-prescribed drugs (one-quarter reported using opioids), only two-thirds of these programs offered overdose education or naloxone distribution and one-third offered fentanyl testing or test strips. This is highly concerning given the high prevalence of fentanyl in both the opioid and non-opioid illicit drug supplies [21] and may partly reflect the presence of policies that criminalize possession of fentanyl test strips in some of the sampled states [22]. Moreover, half of clients who attended MOUD programs without SSP or wound care actively injected drugs. While it is possible that these clients seek safe injection supplies elsewhere, a minority (14%) reported visiting an SSP in the past month.

There were also discrepancies in services offered by SSPs relative to client-reported service utilization. Of clients recruited from SSPs without MOUD, 22% indicated receiving methadone and 8% reported receiving buprenorphine in the past month. This implies clients are either seeking these medications via other service providers or acquiring them on the street, which has been reported to often be easier than enrolling in formal treatment [9, 23, 24]. Roughly half of MOUD programs offered same-day treatment initiation. Additionally, SSP programs were reaching the highest risk population that with the greatest rates of active drug use. Yet, on average, these programs reported having the smallest number of staff and the least available treatment or social services relative to the other programs types. The limited workforce and services offered may reflect the limited budgets often used to operate these programs. Many harm reduction services operate independently from the medical system and are not eligible for insurance reimbursement. Additionally, programs have been historically banned from accessing federal and local funds for SSPs; programs have had to depend on scarce funds acquired a combination of small grants, individual donations, and charitable foundations [4, 25]. The Biden Administration’s 2021 American Rescue Act was the first federal action to allocate targeted funding toward harm reduction services and SSPs [25, 26]. While this was an important step to potentially help scale up these services, local and national resistance and stigma to these programs remains persistent (highlighted by the recent resistance to federal funding sterile pipes [27]). Continued efforts to combat ongoing stigma and political resistance to these programs are needed [25].

Findings from this study demonstrate that in many ways, existing programs are not adequately meeting the service needs of or catering to the realities of PWUD. Creating a substance use service system that is truly person-centered and successful at improving health and dignity will necessitate moving away from the binary mentality of harm reduction vs. treatment to one which is better tailored to individual clients. This includes offering a continuum of co-located treatment, harm reduction, and social services that can meet individuals where they are. This would help facilitate access to life-saving services and greater socioeconomic stability [28, 29]. This may be particularly important for individuals with multiple vulnerabilities, as well as during emergencies—such as the COVID-19 pandemic—when minimizing travel and co-locating access to multiple health and social services is key [30]. In our study, programs that included both MOUD & SSP offered the greatest range of treatment and harm reduction services, including naloxone distribution, overdose prevention education, same-day treatment initiation, drop-in spaces, peer services/street outreach, and counseling services. However, these programs were the rarest in our sample of providers and remain largely under-resourced and at the periphery of the substance use service system. Moreover, such integrated models have been made possible by the ability to prescribe buprenorphine in non-traditional treatment settings [31]. Methadone, which may be the most effective and desirable MOUD option for some individuals, and used by many participants in our study, is still largely restricted to the opioid treatment program system bound by regulations on staffing, zoning, and hefty requirements for patients such as frequent urine drug screening [32, 33]. While there are some successful models of lower threshold methadone in other countries[34], scaling up methadone to meet needs of PWUD in the USA will require rethinking some of the core federal and state regulations, including expanding methadone availability beyond the opioid treatment program system [35]. It is important to note that most participating clients reported using drugs other than opioids; thus, integrating interventions for stimulant and other drug use should be central to efforts to better align programs with client behaviors.

Finally, across all program types, we identified important gaps in social and auxiliary services available relative to the socioeconomic circumstances of clients. While most programs offered on-site or referral to case management and social services, only a minority offered any direct housing support despite nearly half of clients being unstably housed. Education and job training were even less available, though nearly half of clients reported being unemployed. Moreover, roughly half of clients identified as female, and many were of parenting age. Yet, only one-third of programs offered any parenting, childcare, or pregnancy services. Lack of integration of these services highlights a missed opportunity to address social determinants of health that strongly influence overdose risk and overall health and stability among this highly vulnerable population[36, 37]. Increasing grant funding for wrap-around services, as was done via the Ryan White HIV/AIDS Program [38], improving insurance coverage of social services via bundled payments [39], and leveraging flexible funding streams, such as those allocated to states in the aftermath of opioid litigation, [40] may be avenues to assist programs in integrating client social services.

The current study is subject to several limitations. First, our sample of programs and clients may not be generalizable; we recruited the sample from Bloomberg Overdose Initiative states via convenience sampling of providers with preexisting relationships with initiative partners. Thus, it is likely that participating programs more likely represent non-for profit and harm-reduction-oriented service providers and that clients are not representative of the broader sociodemographic or behavioral characteristics of PWUD nationwide. For example, 100% of included MOUD only programs were non-for-profit organizations, while less than 40% of US opioid treatment programs that deliver all three types of MOUD are non-for-profit organizations [41]. Relatedly, clients who participated in this survey may be distinct from treatment and harm reduction clients more broadly and differ characteristically from those who did not choose to participate in the study. Indeed, the majority of clients recruited from SSPs were white, which may reflect the unique geography/demographics of the regions from which programs were recruited, but could also be indicative of greater barriers to accessing harm reduction services among Black and other minoritized groups [42], which should be a subject of future research. Moreover, client survey questions that inquired about treatment and harm reduction service utilization did not distinguish between services accessed at the program from which clients were recruited from versus other service providers and did not explicitly ask clients about their service utilization or treatment goals. Data are also based on cross-sectional surveys conducted during the early months of the COVID-19 pandemic and may not represent typical service provision or utilization and characteristics of clients at other times. Finally, proportion comparisons and p-values across provider characteristics should be interpreted with caution and be considered exploratory due to small sample and cell sizes.

## Conclusions

The enduring overdose crisis intertwined with the ongoing COVID-19 pandemic demonstrates the need to realign health service systems with the needs of the communities they are meant to serve. History of drug use stigma and discrimination has led to a bifurcated approach to delivering services. To address the current and future crises, policymakers, payers, health systems and service providers should increase service integration opportunities, wrap-around funding, and support the spectrum of services related to the health of PWUD. Increased funding and attention to substance use as a part of holistic healthcare, with further investment and use of innovative and individualized client care models, can pave the way for a more effective and equitable service system that promotes the long-term health of people who use drugs.

## Data Availability

The datasets generated and/or analyzed during the current study are not publicly available due to study protocols that prioritize preserving the privacy/anonymity of participants.

## Abbreviations

PWUD: People who use drugs
OUD: Opioid use disorder
MOUD: Medications for opioid use disorder (MOUD)
SSP: Syringe services program
